# Relationship of serum Vitamin D concentrations with Adipokines and Cardiometabolic risk among non-Hispanic black type 2 diabetic and non-diabetic subjects: a cross-sectional study

**DOI:** 10.1186/s40795-018-0259-2

**Published:** 2018-12-18

**Authors:** Janet Antwi, Fatma Huffman, Stacey Sullivan

**Affiliations:** 1 Dietetics and Nutrition, Human Ecology Department, State University of New York at Oneonta, New York, USA; 2 Department of Dietetics and Nutrition, Robert Stempel College of Public Health, Florida International University, Miami, USA

**Keywords:** Serum 25-hydroxyvitamin D [25(OH)D], Vitamin D, Type 2 diabetes, BMI, Leptin, Interleukin-6, Adiponectin, Haitian Americans, African Americans, Adipokines

## Abstract

**Background:**

To report the association of serum 25-hydroxyvitamin D [25(OH)D] and its association with adipokines and cardiometabolic risk factors in Haitian Americans (HA) and African Americans (AA) by ethnicity and type 2 diabetes (T2D) status.

**Methods:**

A cross-sectional study in 197 HA (92 with T2D and 102 without T2D) and 200 AA (97 with T2D and 103 without T2D) recruited in South Florida. Serum 25(OH)D concentrations and adipokines were analyzed by ELISA and cardiometabolic risk factors were indexed by obesity, glycemic control, insulin sensitivity, lipid profile, and blood pressure.

**Results:**

Controlling for age, BMI, energy intake, smoking status and HOMA2-IR in multivariate linear regression analyses, serum 25(OH)D concentrations were significantly associated with WC (R^2^ = 0.760, B = − 0.092, *P* = 0.027), HbA1C (R^2^ = 0.142, B = − 0.012, *P* = 0.010), and TG (R^2^ = 0.159, B = − 1.192, *P* = 0.003) in only HA without T2D. While serum 25(OH)D concentrations were significantly associated with TC (R^2^ = 0.168, B = − 0.329, *P* = 0.040), log leptin (R^2^ = 0.544, B = − 0.007, *P* = 0.021), and adiponectin (R^2^ = 0.144, B = 0.111, *P* = 0.033), but slightly associated with LDL-c (R^2^ = 0.133, B = − 0.278, *P* = 0.064) in only AA without T2D. Among individuals with T2D, serum 25(OH)D concentrations were marginally associated with IL-6 (R^2^ = 0.109, B = 0.076, *P* = 0.085) in HA with T2D, and there was a trend toward significance with log leptin (R^2^ = 0.393, B = − 0.006, *P* = 0.075) in AA with T2D in regression analysis.

**Conclusions:**

The findings that the associations of serum 25(OH)D concentrations with adipokines and cardiometabolic factors differ between HA and AA has clinical and public implications to guide design of T2D preventive strategies that are culturally specific even within the same ethnicity.

**Electronic supplementary material:**

The online version of this article (10.1186/s40795-018-0259-2) contains supplementary material, which is available to authorized users.

## Background

Low vitamin D status as generally indicated by circulating serum 25-hydroxyvitamin D (25(OH)D) concentrations is linked to increased risk of type 2 diabetes (T2D) and cardiovascular diseases [1, 2]. Cross-sectional and observational studies have demonstrated associations between serum 25(OH)D concentrations and cardiometabolic risk factors such as obesity, inflammation, glycemic control, insulin sensitivity, abnormal lipid profile, and high blood pressure (BP), but with inconsistent results [3–7]. Additionally, it is postulated that vitamin D may regulate systemic inflammation through immunomodulatory actions on several peptides known as adipokines to improve glycemic control, insulin sensitivity, and endothelial function [8–11].

Previous research has found that adipokines including leptin, adiponectin, and interleukin-6 (IL-6) regulate glucose and lipid metabolism, vascular remodeling, and are involved in inflammatory responses [12–17]. Fontana et al. demonstrated that a low-grade inflammation observed with circulating levels of leptin and IL-6 is directly proportional to the body fat mass, particularly central adiposity as measured by waist circumference (WC). In contrast, adiponectin is inversely associated with body fat and has an anti-inflammatory effect, a protective action for cardiometabolic health. One randomized controlled trial revealed that low serum 25(OH)D concentrations were found to be associated with increased leptin, and decreased adiponectin levels in the treatment group as compared to the control group [12] However, a cross-sectional analysis conducted by Almetwazi et al. using the 2003–2006 National Health and Nutrition Examination Survey (NHANES) sample of 929 diabetic adults aged 18 years or older found no association between serum 25(OH)D concentrations and hemoglobin A1C (HbA1C) levels, after adjustment for race/ethnicity, BMI, age, gender, type of diabetic medication used, having health insurance or not, and comorbid conditions (hypertension, anemia, cholesterol, liver disease, and kidney disease) [18]. Furthermore, Kim et al. studied the relationship between serum 25(OH)D concentrations and the risk of cardiovascular disease predictors. They found that after controlling for factors that may affect the cardiovascular index (age, sex, body mass index, smoking, and alcohol intake), serum 25(OH)D concentrations was related to triglyceride, HDL-cholesterol but not to IL-6 [19]. Recent studies investigating a relationship between serum 25(OH)D concentrations and serum lipids as measured by total cholesterol (TC), low density lipoprotein cholesterol (LDL-c) triglycerides (TG), and high density lipoprotein cholesterol (HDL-c) have been controversial [20, 21]. Although these prior studies have been valuable, they have been limited comparatively by low representation from non-Hispanic blacks. Further, the variability in the results of these studies may be likely due to variations in geographical location and differences in the populations studied.

Epidemiological research has established that both lower circulating 25(OH)D and lower intakes of vitamin D are more common in non-Hispanic blacks than in non-Hispanic whites. This is important because ethnic differences also exist in T2D such that blacks have high prevalence of cardiometabolic risk factors such as large WC, inflammation, lower insulin sensitivity and hypertension, with greater risk of T2D compared to non-Hispanic whites [22, 23]. Interestingly, the association of 25(OH)D with T2D was found to differ even within the same non-Hispanic black ethnicity. Shaban et al. [24] observed that African Americans (AA) had lower serum 25(OH)D concentrations associated with higher odds of having T2D than Haitian Americans (HA). Moreover, HA had the lowest body mass index (BMI), WC, higher insulin sensitivity, and lower insulin resistance, but the associations of these parameters with serum 25(OH)D concentrations were not investigated. The relationship of serum 25(OH)D concentrations with adipokines and several cardiometabolic risk factors has been far less been investigated within the non-Hispanic black ethnicity. Given the differences in cardiometabolic health between HA and AA, it is crucial to examine the relationship of serum 25(OH)D concentrations with adipokines and cardiometabolic risk factors within the subgroups of non-Hispanic blacks. A better understanding of the intra-ethnic differences of such associations may help explain and inform future research into the pathophysiology of serum 25(OH)D concentrations in cardiometabolic health in the non-Hispanic black ethnic group.

Accordingly, we examined if the association of serum 25(OH)D concentrations with adipokines, central adiposity, glycemic control, insulin sensitivity, lipid profile, and BP, independent of demographic variables, total body adiposity (BMI) and insulin resistance will differ between HA and AA type 2 diabetic and non-diabetic community dwellers in a year-round sunny climate in South Florida.

## Methods

### Study population

This cross-sectional study was conducted among 197 HA (92 with T2D and 102 without T2D) and 200 AA (97 with T2D and 103 without T2D). Study participants were recruited from Miami- Dade and Broward Counties, in South Florida. Recruitment of African American participants was based on response to flyers sent from random selection of randomly generated mailing lists that were purchased from Knowledge Base Marketing, Inc., Richardson, TX, USA. This company provided two mailing lists generated from multiple databases of African Americans identified as having or not having T2D. About 7550 letters were mailed to African Americans with and without T2D to request their participation. Of the letters mailed, approximately 6.3% (*n* = 477) were returned due to unknown addresses, while 4% (*n* = 256) of the remaining delivered letters responded. Because of unavailability of a similar database of mailing addresses for Haitian Americans, multiple community-based sources were used to recruit Haitian Americans (*n* = 259). Print advertisements were posted in local Haitian supermarkets, churches, and restaurants; flyers were also distributed to Florida International University (FIU) faculty, staff and students. In addition, local diabetes educators and community health professionals in Miami-Dade and Broward counties were requested to assist in recruitment efforts, and announcements were also aired on a Creole radio station. Interested individuals were interviewed on phone and were fully informed of the study purpose. For all those interviewed, age (≥35 years), gender, and self-identified ethnicity (HA or AA) were assessed. For respondents who self-reported as having T2D, initial treatment modalities, duration of diabetes and fasting plasma glucose (FPG) and HbA1C were obtained. Exclusion criteria included kidney failure, hepatitis, human immunodeficiency virus (HIV) and cancer. Eligible participants were requested to enroll in the study at the Human Nutrition Laboratory at Florida International University (FIU). Participants to enroll in the study were instructed to fast for at least 8 h prior to their blood collection. Additionally, they were directed to refrain from smoking and any unusual exercise, but could drink only water. Twelve participants (HA = 8; AA = 4) were reclassified as having T2D according to the criteria specified by the American Diabetes Association and referred to their physicians. Written informed consent in English or Creole was obtained from each participant after they understood the requirements on the form. The study protocol was approved by the Institutional Review Board at FIU.

### Sociodemographic data

A standardized self-reported questionnaire was used to obtain information regarding age, gender, smoking status, and medications used. Self-reported information on ethnicity was confirmed during interview.

### Anthropometric measurement

Height and weight were measured to the nearest 0.1 cm and 0.1 kg respectively, using a SECA balance scale (Seca Corp, Columbia, MD). BMI was calculated as weight in kg/height in m^2^. The cutoff point for obesity was BMI ≥ 30 kg/m^2^. Waist circumference (WC) was measured to the nearest 0.1 cm with a non-stretchable measuring tape, midway between the lower rib margin and the iliac crest. Blood pressure [systolic (SBP) and diastolic (DBP)] was measured twice with a sphygmomanometer (Tycos 5090–02 Welch Allyn Pocket Aneroid Sphygmomanometer, Arden, NC, USA) and a stethoscope (Littmann Cardiology, 3 M, St Paul, MN, USA) and averaged with the participants in a sitting position at rest for fifteen minutes.

### Assessment of dietary intake

The validated and standardized Harvard semi-quantitative food frequency questionnaire was used to obtain information on macro- and micro-nutrients from foods and vitamins consumed. Participants self-reported average intake of specified quantities of foods over the past year. Daily servings for food groups were calculated by totaling frequency scores for all food items. The output of these calculations was used to obtain the energy intake (kcal) of participants.

### Blood collection and laboratory measurements

Twenty milliliters of venous blood was collected in a Vaccutainer Serum Separator tubes by a trained and certified phlebotomist using standard methods, following an 8-h overnight fast. Blood samples were centrifuged at 2500 RPM for half an hour after coagulation. The separated serum was aliquoted into 3 labeled plastic tubes. One was designated for analysis of serum 25(OH)D. The third aliquot was stored at -70 °C for subsequent analyses. Glycated hemoglobin levels were assayed in whole blood samples applying the Roche Tina Quant method with both mean intra-assay and inter-assay coefficient of variation (CV) of 3.5% (Laboratory Corporation of America, FL, USA). Serum lipid levels (TC, LDL-c, HDL-c, and TG) were determined by automatic chemical analyzer (Laboratory Corporation of America, FL, USA). Serum 25(OH)D concentrations were determined with an ELISA kit with both mean intra-assay and inter-assay CV of less than 10.3% from Immunodiagnostic Systems Limited (Scottsdale, AZ, USA). Serum adipokines and inflammatory markers were measured by ELISA kits. The mean intra-assay and inter-assay CV of leptin levels were 3.0 and 4.2%, respectively with assay sensitivity of 7.8 pg/ml (R&D Systems, Minneapolis, MN, USA). For IL-6, both mean intra-assay and inter-assay CV were less than 10% with assay sensitivity of 2.2 pg/ml (BD Biosciences, San Jose, CA, USA). Serum adiponectin had intra-assay and inter-assay CV as 7.4 and 8.4% (Linco Research Inc., MO, USA).

### Calculation of insulin sensitivity (HOMA2-IS) and insulin resistance (HOMA2-IR)

The Oxford University homeostasis model assessment version 2 (HOMA2) calculator was used to calculate homeostasis model assessment version 2 of insulin sensitivity index (HOMA2-IS) and homeostasis model assessment version 2 of insulin resistance index (HOMA2-IR) as previously described [25]. The model determines HOMA2-IS and HOMA2-IR from paired fasting plasma glucose and radioimmunoassay insulin across a range of 1–2200 pmol/L for insulin, and 1–25 mmol/L for glucose.

### Data analysis

Cross-sectional data analyses were conducted with SPSS version 21 (IBM Corporation, Chicago, IL, USA). The Kolmogorov-Smirnov test was used to assess the normality of the distribution of the data. The distributions of leptin and HOMA2-IR were natural log-transformed to improve normality. Descriptive statistics were computed and presented as mean ± SD for continuous variables, and frequencies (percentages) for categorical variables. Differences in mean values for each ethnic subgroup by diabetes status were compared using Student’s t-test for continuous variables and χ^2^ tests for categorical variables. Partial Pearson’s correlation coefficients were used to examine the associations between serum 25(OH)D concentrations and cardiometabolic risk factors with adjustment for age, BMI, energy intake and log HOMA2-IR. Multivariate linear regression (MLR) analyses were conducted to determine the contributions of serum 25(OH)D concentrations to cardiometabolic risks factors after adjusting for the same covariates, including smoking status. For all analyses, a *P*-value of < 0.05 was considered to be statistically significant.

## Results

Participant clinical characteristics by ethnicity and diabetes status are shown in Table 1. There were significant differences of most study characteristics between individuals with and without T2D in AA, but not in HA. African Americans with T2D had lower serum 25(OH)D levels (*P* = 0.001) and lower vitamin D intake (*P* = 0.016) than AA without T2D. African Americans with T2D also had higher BMI and larger WC (*P* < 0.001 for both), as compared to AA without T2D. No statistically significant differences in these characteristics were found between HA with and without T2D. The levels of log HOMA2-IR (*P* = 0.030), HOMA2-IS (*P* = 0.021), SBP (*P* = 0.003), IL-6 (*P* = 0.049), and adiponectin (*P* = 0.032) were statistically different between AA with and without T2D, but these differences were not observed among the HA ethnic subgroup. Haitian Americans with T2D had lower LDL-c (*P* = 0.029) than HA without T2D, whereas such difference was not revealed in AA. The older age (*P* = 0.005 for HA and *P* = 0.008 for AA), lower energy intake (*P* = 0.004 for HA and *P* = 0.002 for AA), and higher HbA1C (*P* < 0.001 for HA and AA) in individuals with T2D than without T2D was detected in the two ethnicities. There were no statistically significant differences in smoking status, TC, HDL-c, TG, DBP, and log leptin levels, between individuals with and without T2D in HA and AA ethnic subgroups.

**Table 1:** Characteristics of study participants of each ethnic subgroup by diabetes status

Variables	Haitian Americans			African Americans		
	without T2D (*n* = 102)	with T2D (*n* = 95)	*P*-value	without T2D (*n* = 103)	with T2D (*n* = 97)	*P*-value
Age (years)	54.1 ± 10.6	58.3 ± 9.9	0.005	51.1 ± 8.3	54.6 ± 10.1	0.008
Gender F (%)	51 (50.0)	51 (53.7)	0.605	51 (49.5)	47 (48.5)	0.881
Smoke n (%)	6 (5.9)	5 (5.3)	0.850	45 (43.7)	36 (37.1)	0.344
Energy intake kcal (kcal/d)	2042.0 ± 1205.0	1589.2 ± 968.9	0.004	2843.6 ± 1971.6	2106.1 ± 1341.1	0.002
BMI (kg/m^2^)	29.2 ± 5.1	29.1 ± 4.4	0.875	30.9 ± 6.0	35.4 ± 8.2	< 0.001
Obese (BMI ≥30 kg/m^2^) (%)	41 (40.2)	41 (43.2)	0.673	56 (54.4)	72 (74.2)	0.030
WC (cm)	96.8 ± 12.8	99.2 ± 11.4	0.172	101.4 ± 14.0	113.9 ± 17.2	< 0.001
HbA1C (%)	6.1 ± .8	8.4 ± 2.6	< 0.001	5.9 ± .5	7.7 ± 2.0	< 0.001
log HOMA2-IR	0.2 ± .5	0.1 ± .6	0.432	0.3 ± .7	0.5 ± .7	0.030
HOMA2-IS	95.7 ± 51.7	102.5 ± 53.4	0.367	91.2 ± 54.8	74.7 ± 44.7	0.021
TC (mg/dL)	199.1 ± 42.5	188.3 ± 39.1	0.067	192.4 ± 40.0	186.0 ± 40.6	0.265
LDL-c (mg/dL)	125.3 ± 40.6	113.6 ± 33.0	0.029	117.5 ± 36.8	111.1 ± 37.9	0.226
HDL-c (mg/dL)	52.0 ± 14.1	54.0 ± 15.4	0.337	51.6 ± 13.9	49.4 ± 13.5	0.262
TG (mg/dL)	108.8 ± 64.6	103.3 ± 52.3	0.515	116.4 ± 57.3	127.6 ± 65.0	0.200
SBP (mmHg)	145.6 ± 24.2	146.3 ± 26.2	0.840	133.6 ± 17.8	141.4 ± 19.3	0.003
DBP (mmHg)	90.7 ± 12.4	89.9 ± 13.9	0.686	88.8 ± 13.0	89.5 ± 11.3	0.667
log Leptin (pg/mL)	9.8 ± .9	9.7 ± 1.2	0.221	9.9 ± 1.0	10.1 ± .8	0.157
IL-6 (pg/mL)	4.4 ± 6.7	5.9 ± 8.9	0.168	7.5 ± 5.6	10.9 ± 16.7	0.049
Adiponectin (ng/mL)	17.1 ± 10.8	16.6 ± 9.8	0.745	22.8 ± 12.8	27.7 ± 18.5	0.032
Vitamin D intake (IU/d)	405.5 ± 387.3	321.3 ± 284.9	0.085	584.9 ± 470.5	440.5 ± 361.7	0.016
25(OH)D (nmol/L)	53.7 ± 16.3	56.8 ± 21.3	0.255	59.9 ± 24.4	48.8 ± 23.1	0.001

Data were expressed as mean ± SD unless otherwise indicated. Abbreviations: *T2D* type 2 diabetes; *BMI* body mass index; *WC* waist circumference; *HbA1C* hemoglobin A1C; *HOMA2-IR* homeostasis model assessment version 2 of insulin resistance; *HOMA2-IS* homeostasis model assessment version 2 of insulin sensitivity; *TC* total cholesterol; *LDL-c* low density lipoprotein cholesterol; *HDL-c* high density lipoprotein cholesterol; *SBP* systolic blood pressure; *DBP* diastolic blood pressure; *IL-6* interleukin 6; 25(OH)D 25-hydroxyvitamin D. *P*-value is significant at < 0.05.

Tables 2 and 3 report the correlations of serum 25(OH)D concentrations with cardiometabolic risk factors. Prior to adjustments, serum 25(OH)D concentrations were correlated negatively with WC, HbA1C and TG in HA without T2D only. While different relationships were observed in AA. Serum 25(OH)D concentrations were correlated negatively with BMI, log HOMA-IR, TC, LDL-c, and log leptin, but positively with HOMA2-IS, and adiponectin in AA without T2D, and positively with HOMA2-IS, but negatively with log leptin in AA with T2D. After controlling for age and BMI in Model 1, all the variables remained significant in HA without T2D, in addition to IL-6 for HA with T2D. However, the correlations with log HOMA2-IR, and LDL-c in AA without T2D, and with HOMA2-IS in both AA with and without T2D disappeared. After further controlling for age, energy intake, and log HOMA2-IR in Model 2, the strong negative correlations in the unadjusted model still kept significant for all variables in HA without T2D. Moreover, log leptin, adiponectin, TC, and LDL-c in AA without T2D, and log leptin in AA with T2D remained correlated.

**Table 2:** Correlations of serum 25(OH)D with different cardiometabolic risk factors in Haitian Americans by diabetes status

Variables	Haitian Americans without T2D (*n* = 102)			Haitian Americans with T2D (*n* = 95)		
	Unadjusted	Model 1	Model 2	Unadjusted	Model 1	Model 2
BMI (kg/m^2^)	− 0.152	–	− 0.133	− 0.175	–	− 0.195
WC (cm)	− 0.229*	− 0.210*	− 0.241*	− 0.119	0.037	− 0.146
HbA1C (%)	− 0.212*	− 0.218*	− 0.215*	0.034	0.031	0.023
log HOMA2-IR	− 0.054	0.008	–	0.104	0.116	–
HOMA2-IS	0.071	0.005	0.034	−0.137	− 0.154	− 0.124
TC (mg/dL)	− 0.053	− 0.046	− 0.039	− 0.170	−0.159	− 0.201
LDL-c (mg/dL)	−0.001	0.012	0.018	− 0.176	−0.151	− 0.195
HDL-c (mg/dL)	0.141	0.119	0.142	0.000	−0.044	− 0.009
TG (mg/dL)	− 0.323**	− 0.317**	− 0.330**	− 0.078	−0.048	− 0.120
SBP (mmHg)	−0.058	− 0.061	−0.080	0.110	0.065	0.078
DBP (mmHg)	−0.066	−0.036	− 0.060	0.045	0.059	0.023
log Leptin (pg/mL)	0.026	0.101	0.032	− 0.096	− 0.004	− 0.092
IL-6 (pg/mL)	− 0.114	− 0.114	− 0.126	0.189	0.207*	0.172
Adiponectin(ng/mL)	0.061	− 0.036	− 0.009	0.147	0.121	0.140

Model 1, adjusted for age and BMI.

Model 2, adjusted for age, energy intake, and HOMA-IR.

^*^P < 0.05,

^**^P < 0.01

**Table 3:** Correlations of serum 25(OH)D with different cardiometabolic risk factors in African Americans by diabetes status

Variables	African Americans without T2D (*n* = 103)			African Americans with T2D (*n* = 97)		
	Unadjusted	Model 1	Model 2	Unadjusted	Model 1	Model 2
BMI (kg/m^2^)	− 0.215*	–	− 0.141	−0.270	–	− 0.175
WC (cm)	−0.136	0.076	− 0.059	− 0.182	0.114	− 0.090
HbA1C (%)	− 0.172	− 0.105	−0.140	− 0.067	0.009	0.015
log HOMA2-IR	− 0.211*	− 0.139	–	− 0.190	−0.130	–
HOMA2-IS	0.199*	0.124	0.008	0.208*	0.150	0.071
TC (mg/dL)	− 0.227*	− 0.196*	− 0.239*	− 0.185	−0.172	− 0.164
LDL-c (mg/dL)	− 0.208*	− 0.172	− 0.219*	− 0.157	−0.133	− 0.118
HDL-c (mg/dL)	−0.056	− 0.094	−0.119	0.028	− 0.063	− 0.097
TG (mg/dL)	− 0.059	−0.008	0.015	− 0.147	− 0.090	− 0.078
SBP (mmHg)	−0.044	− 0.005	−0.016	− 0.096	−0.163	− 0.112
DBP (mmHg)	−0.163	− 0.080	− 0.115	− 0.179	−0.158	− 0.117
log Leptin (pg/mL)	− 0.303**	− 0.232*	− 0.265**	− 0.282**	− 0.213*	− 0.249*
IL-6 (pg/mL)	0.123	0.133	0.133	−0.171	− 0.151	−0.133
Adiponectin(ng/mL)	0.255**	0.231*	0.215*	0.046	−0.087	− 0.068

Model 1, adjusted for age and BMI.

Model 2, adjusted for age, energy intake, and HOMA-IR.

^*^P < 0.05,

^**^P < 0.01

In MLR analyses within each ethnic subgroup and by diabetes status, serum 25(OH)D concentrations significantly explained the variances in WC (R^2^ = 0.760, B = − 0.092, *P* = 0.027), HbA1C (R^2^ = 0.142, B = − 0.012, *P* = 0.010), and TG (R^2^ = 0.159, B = − 1.192, *P* = 0.003) in only HA without T2D. While in AA without T2D only, serum 25(OH)D concentrations significantly explained the variances in TC (R^2^ = 0.168, B = − 0.329, *P* = 0.040), log leptin (R^2^ = 0.544, B = − 0.007, *P* = 0.021), and adiponectin (R^2^ = 0.144, B = 0.111, *P* = 0.033), but slightly associated with LDL-c (R^2^ = 0.133, B = − 0.278, *P* = 0.064), after adjustment for previously stated covariates. However, among individuals with T2D, serum 25(OH)D concentrations were marginally associated with IL-6 (R^2^ = 0.109, B = 0.076, *P* = 0.085) in HA with T2D, and there was a trend toward significance in the association with log leptin (R^2^ = 0.393, B = − 0.006, *P* = 0.075) in AA with T2D in regression analysis.

## Discussion

This study examined the relationships of serum 25(OH)D concentrations with various cardiometabolic risk factors across diabetes status within non-Hispanic black ethnic subgroups living in a sun-rich climate. Significant differences across diabetes status within the ethnic subgroups were found. Specifically, there were significant differences of serum 25(OH)D concentrations between individuals with and without T2D in AA, but not in HA. Results indicated that, serum 25(OH)D concentrations were inversely associated with WC, HbA1C, TG levels specific for HA without T2D, whereas inversely associated with TC, and leptin, and positively associated with adiponectin in AA without T2D only. To our knowledge, apart from the study [24] mentioned that determined few risk factors and did not explore associations with serum 25(OH)D concentrations, this is the only study that has included several cardiometabolic risk factors and their relationships with serum 25(OH)D concentrations adjusted for important confounders in HA and AA with and without T2D residing in sunny locations in the U.S.

African Americans with T2D had the lowest serum 25(OH)D concentrations, as compared to AA without T2D, a finding not observed in HA, indicative of an ethnic difference. A possible explanation to this observation in the present study may be due to AA with T2D having the highest BMI, largest WC and highest percentage obese than AA without T2D. However, differences in BMI, WC and percentage obese in HA with and without T2D was not significantly different. Links between vitamin D deficiency and adiposity have been well established [26, 27]. The mechanisms proposed for the inverse relationship of serum 25(OH)D with BMI and WC are the sequestration of vitamin D in the increased fat mass reducing release into systemic circulation, and dilution of ingested or cutaneously synthesized vitamin D by the large body fat compartments reducing bioavailability [27–29]. Additionally, several studies have shown that low serum 25(OH)D could in turn lead to obesity, because its influence on free fatty acids mobilization in the adipose tissue as well as metabolism of fat cell which lead to increases in energy expenditure, a mechanism of weight loss may be downgraded [30–32]. Thus, the lower intake of vitamin D coupled with lower serum 25(OH)D in AA with T2D than in AA without T2D may explain the higher BMI in AA with T2D than AA without T2D in spite of lower energy intake. It is noteworthy to mention that, the higher vitamin D intake in AA without T2D was not because of dietary supplementation, thus it could be attributed to the increased energy intake in this sample of AAs. Previous studies have found that AA normally have low intakes of vitamin D since most of the foods fortified with vitamin D (e.g., milk and dairy products) are lactose-containing to which AA are intolerant due to lactase deficiency commonly found in this population [33]. Vitamin D intake was not significantly different between HA with and without T2D. It is also not unexpected that in regression analyses, serum 25(OH)D concentrations were associated with WC in HA without T2D, but not in AA without T2D. The reason for the discrepancy may be due to the high serum 25(OH)D concentrations as well as high vitamin D intake in AA without T2D, since the entire study sample was made up of mainly overweight and obese individuals. Considering reduced production of endogenous vitamin D through sunlight secondary to greater melanin pigmentation [34], dietary vitamin D intake is crucial in this ethnic group with regards to obesity and its comorbidities.

Our study adds to the literature that serum 25(OH)D has direct and indirect effects on β-pancreatic cells promoting insulin signaling and secretion, thus play an important role in insulin sensitivity, insulin resistance, and possibly glycemic control [35–37]. There was a significant association between 25(OH)D and HOMA2-IR in AA without T2D, and with HOMA2-IS in both AA with and without T2D. However, these relationships attenuated after adjustment for age and BMI in correlation analyses. Moreover, after adjusting for age, energy intake, and HOMA2-IR the negative association between serum 25(OH)D and BMI in AA without T2D only, became not significant. This bolsters the evidence of the interconnectedness between age, energy intake, BMI, serum 25(OH)D and glucose metabolism [3–5]. It is suggested that increasing age, BMI and energy intake not only decrease the effectiveness of insulin signaling pathways that result in decreased insulin sensitivity and increased insulin resistance, but also affect serum 25(OH)D levels. Thus, controlling for the interactions and influence of these covariates in the relationship of serum 25(OH)D with HOMA2_IR and HOMA2-IS may have caused the significant results to disappear. Although HA were older, they had higher insulin sensitivity and lower insulin resistance and so these associations previously discussed were not seen in them, suggesting that there are other factors that may have a protective role in HA. For example, HA have been found to have better diet quality, lifestyle factors, and different genetic factors than AA [38–40]. The association between serum 25(OH)D concentration and HbA1C was observed in HA without, T2D only. It is not clear why the relationship between serum 25(OH)D and HbA1C would be specific for HA despite having better cardiometabolic metabolic parameters. One reason could be that HA have been found to have poorer control of their HbA1C levels compared to AA [41] which we observed in the current study that HA had higher HbA1C levels regardless of diabetes status. Further carefully controlled experimental study is needed to better understand the observation.

Low serum 25(OH)D concentrations have been associated with dyslipidemia, indicated as increased serum levels of TC, LDL-c and TG, and low levels of HDL-c, which could possibly explain the relation with diabetes and cardiovascular diseases [42, 43]. Ford et al. [44] in their analysis of the third National Health and Nutrition Examination Survey (NHANES) revealed inverse associations between serum 25(OH)D concentrations and TG in healthy subjects. Moreover, Karhapää et al. [45] reported that serum 25(OH)D concentrations were negatively associated with TC, LDL-c and TG and positively with HDL-c after controlling for age and BMI. Both HA and AA in our sample had high levels of TC, LDL-c and TG, with lower levels of HDL-c observed mostly in AA. In theory, vitamin D is thought to affect lipid levels directly by maintaining adequate amounts of apolipoprotein A-1, a major component of HDL cholesterol needed to clear LDL-c and TG from circulation [46]. In addition, it was suggested that the effect of vitamin D in decreasing serum levels of LDL-c and TG may occur through modulatory action that increases the activity of lipoprotein lipase in adipose tissue [47]. The indirect effects of vitamin D on lipids could be through calcium formation of insoluble complexes with saturated fats and thus reduction in serum levels of TC and LDL-c [48]. The statistically significant negative relationships between serum 25(OH)D concentrations and TG in HA without T2D, but with TC and marginally with LDL-c in AA without T2D we found in our sample, independent of BMI suggest the differing associations may explain, in part, ethnic differences in cardiometabolic health. Vitamin D intervention studies comparing outcomes in both HA and AA are required to confirm the ethnic differences.

We observed that independent of BMI, the association of serum 25(OH)D concentrations with adipokines were more pronounced in AA than in HA. Serum 25(OH)D concentrations were significantly inversely related with leptin levels and positively with adiponectin in AA without T2D, while a marginal inverse association with IL-6 in HA without T2D was observed. However, the reasons for the discordance in the specific types of adipokines related to 25(OH)D between HA and AA may be due to differences in the levels of these adipokines and other cardiometabolic profile between the two ethnic subgroups. Studies investigating all forms and sources of vitamin D and their relationships with robust measures of these adipokines in the Black ethnic subgroup is required to further understand the causal role of vitamin D on adipokines and inflammatory processes. The significant relation of 25(OH)D with leptin and adiponectin was found in a study performed in a healthy adult population, low 25(OH)D correlated with increased leptin levels and decreased adiponectin levels [49–51]. The lack of significant association between 25(OH)D and IL-6 was also found in the Framingham adult subjects [52]. Several mechanisms of how vitamin D affects adipokines concentrations have been proposed. It is suggested that vitamin D receptors in the adipocytes of the adipose tissue may regulate adipokine gene expression and vitamin D deficiency may be associated with increased leptin levels and decreased adiponectin levels [53].

It is important to note that although the observed positive and negative associations between serum 25(OH)D concentrations and the cardiometabolic risk factors discussed in HA and AA without T2D were also seen in those with T2D [data not shown (see Additional file 1)], the results were not significant. The slight trend towards significance for adipokines IL-6 in HA with T2D, and log leptin in AA with T2D shows the important role that 25(OH)D plays to regulate other cardiometabolic processes through adipokines in low-grade inflammation [8–11]. A possible explanation for the non-significant results in T2D may be the similarities in general or even better in individuals with T2D in some of the parameters such as BMI, WC, lipids, BP and adipokines for which makes it difficult to detect significant differences between those with and without T2D. Another explanation may be due to the residual confounding effects of medications used mainly by participants with T2D and the relationship between serum 25(OH)D concentrations and HbA1C, lipids, and adipokines may be affected by disease duration in individuals with T2D.

The strength of our study is that we are the first to report differences in the association between serum 25(OH)D concentrations and WC, HbA1C, TG, TC, leptin and adiponectin within non-Hispanic black ethnic subgroups with and without T2D living at Southern U.S. Florida after controlling for age, BMI, smoking status, energy intake and HOMA2-IR. Thus, this study underscores the need to target these non-Hispanic black ethnic subgroups separately considering the profound differences in cardiometabolic risk factors. Apart from using it to control for smoking status, we chose to conduct MLR after partial correlations analyses to compare significant effect modification of associations within non-Hispanic black ethnic subgroups by diabetes status and we reported the output of both analyses to highlight these potential differences discussed above. Nevertheless, the cross-sectional study design of our study limits us from establishing causality between the effects of serum 25(OH)D concentrations on those cardiometabolic risk factors in HA and AA with and without T2D. Longitudinal studies are needed to address this issue. Although this study used HOMA2-IS and HOMA2-IR an estimate of insulin sensitivity and insulin resistance, respectively rather than the gold standard of hyperinsulinemic-euglycemic clamp, the HOMA2-IS and HOMA2-IR have been used in several cross-sectional studies and have been shown to be a reliable surrogate in determining measures of insulin resistance [54]. The semi-convenience nature of our sample is not representative of HA and AA and thus, limits generalizability to the larger population.

## Conclusion

We demonstrated that low serum 25(OH)D concentrations were related with various cardiometabolic risk factors in HA and AA with and without T2D living in a sunny climate all year in the U.S., findings not attributable to adiposity and geographical location. These findings have important clinical and public health implications. Given the high prevalence of vitamin D deficiency and ethnic disparity of these associations observed even within the same Black ethnicity, additional prospective studies or well-designed randomized clinical trials are urgently needed to help characterize the ethnic differences in the relationship between vitamin D status, obesity, inflammation, glycemic control and dyslipidemia to prevent T2D in this population.

## Additional file

Additional file 1: Haitian Americans and African Americans with T2D – (DOCX 12.3 kb)

## Abbreviations

AA: African Americans.
BMI: Body mass index.
BP: High blood pressure.
DBP: Diastolic blood pressure.
[25(OH)D]: Serum 25-hydroxyvitamin D.
ELISA: Enzyme-linked immunosorbent assay
FIU: Florida International University.
FPG: Fasting plasma glucose.
HA: Haitian American.
HbA1C: Hemoglobin A1C.
HDL-c: High density lipoprotein cholesterol.
HIV: Human Immunodeficiency virus.
HOMA2-IR: Homeostasis model assessment version 2 of insulin resistance index.
HOMA2-IR: Homeostasis model assessment version 2 of insulin sensitivity index.
HOMA2: Homeostasis model assessment version 2.
IL-6: Interleukin-6.
LDL-c: Low density lipoprotein cholesterol.
MLR: Multivariate linear regression.
NHANES: National Health and Nutrition Examination Survey.
SBP: Systolic blood pressure.
TC: Total cholesterol.
T2D: Type 2 diabetes.
TG: Triglycerides.
WC: Waist circumference.

## References

[1] Palomer X, González-Clemente JM, Blanco-Vaca F, Mauricio D (2008). Role of vitamin D in the pathogenesis of type 2 diabetes mellitus. Diabetes Obes Metab.

[2] Zittermann A (2006). Vitamin D and disease prevention with special reference to cardiovascular disease. Prog Biophys Mol Biol.

[3] Need AG, O'Loughlin PD, Horowitz M, Nordin BE (2005). Relationship between fasting serum glucose, age, body mass index and serum 25 hydroxyvitamin D in postmenopausal women. Clin Endocrinol.

[4] Borissova AM, Tankova T, Kirilov G, Dakovska L, Kovacheva R (2003). The effect of vitamin D3 on insulin secretion and peripheral insulin sensitivity in type 2 diabetic patients. Int J Clin Pract.

[5] Heshmat R, Tabatabaei-Malazy O, Abbaszadeh-Ahranjani S (2012). Effect of vitamin D on insulin resistance and anthropometric parameters in type 2 diabetes; a randomized double-blind clinical trial. DARU Journal of Pharmaceutical Sciences.

[6] Forman JP, Giovannucci E, Holmes MD, Bischoff-Ferrari HA, Tworoger SS, Willett WC, Curhan GC (2007). Plasma 25-hydroxyvitamin D levels and risk of incident hypertension. Hypertension.

[7] Judd S, Tangpricha V, Deficiency VD (2008). Risk for cardiovascular disease. Circulation.

[8] Kardas F, Kendirci M, Kurtoglu S (2013). Cardiometabolic risk factors related to Vitamin D and adiponectin in obese children and adolescents. Int J Endocrinol.

[9] Roth CL, Elfers C, Kratz M, Hoofnagle AN (2011). Vitamin D deficiency in obese children and its relationship to insulin resistance and adipokines. J Obes.

[10] Krivošíková Z, Gajdoš M, Šebeková K (2015). Vitamin D levels decline with rising number of Cardiometabolic risk factors in healthy adults: association with Adipokines, inflammation, oxidative stress and advanced glycation markers. PLoS One.

[11] Gannagé-Yared MH, Chedid R, Khalife S, Azzi E, Zoghbi F, Halaby G (2009). Vitamin D in relation to metabolic risk factors, insulin sensitivity and adiponectin in a young middle-eastern population. Eur J Endocrinol.

[12] Breslavsky A, Frand J, Matas Z, Boaz M, Barnea Z, Shargorodsky M (2013). Effect of high doses of vitamin D on arterial properties, adiponectin, leptin and glucose homeostasis in type 2 diabetic patients. Clin Nutr.

[13] Fontana L, Eagon JC, Trujillo ME, Scherer PE, Klein S (2007). Visceral fat adipokine secretion is associated with systemic inflammation in obese humans. Diabetes.

[14] Weyer C, Funahashi T, Tanaka S, Hotta K, Matsuzawa Y, Pratley RE, Tataranni PA (2001). Hypoadiponectinemia in obesity and type 2 diabetes: close association with insulin resistance and hyperinsulinemia. J Clin Endocrinol Metab.

[15] Ismail MM, Abdel Hamid TA, Ibrahim AA, Marzouk H (2017). Serum adipokines and vitamin D levels in patients with type 1 diabetes mellitus. Arch. Med. Sci.

[16] Giulietti A, van Etten E, Overbergh L, Stoffels K, Bouillon R, Mathieu C (2007). Monocytes from type 2 diabetic patients have a pro-inflammatory profile. 1,25-Dihydroxyvitamin D(3) works as anti-inflammatory. Diabetes Res Clin Pract.

[17] Hattori A, Uemura K, Miura H, Ueda M, Tamaya N, Iwata F, Muraguchi M, Ohmoto Y, Iguchi A (2000). Gender-related difference in relationship between insulin resistance and serum leptin level in Japanese type 2 diabetic and non-diabetic subjects. Endocr J.

[18] Almetwazi MS, ONoor A, Almasri DM, Popovici I, Alhawassi T, Alburikan KA, Harrington CA (2017). The association of vitamin D deficiency and glucose control among diabetic patients. Saudi Pharm J.

[19] Kim M, Na W, Sohn C (2013). Correlation between vitamin D and cardiovascular disease predictors in overweight and obese Koreans. J Clin Biochem Nutr.

[20] Saedisomeolia A, Taheri E, Djalali M, Moghadam AM, Qorbani M (2014). Association between serum level of vitamin D and lipid profiles in type 2 diabetic patients in Iran. J Diabetes Metab Disord.

[21] Jorde R, Grimnes G (2011). Vitamin D and metabolic health with special reference to the effect of vitamin D on serum lipids. Prog Lipid Res.

[22] Scragg R, Sowers M, Bell C (2004). Serum 25-hydroxyvitamin D, diabetes, and ethnicity in the third National Health and nutrition examination survey. Diabetes Care.

[23] Christensen MHE, Scragg RK (2016). Consistent ethnic specific differences in diabetes risk and vitamin D status in the National Health and nutrition examination surveys. J Steroid Biochem Mol Biol.

[24] Shaban LH, Zarini GG, Exebio JC, Sukhram SD, Huffman FG (2012). Serum vitamin D insufficiency and diabetes status in three ethnic minority groups. J Immigr Minor Health.

[25] Wallace TM, Levy JC, Matthews DR (2004). Use and abuse of HOMA modeling. Diabetes Care.

[26] Cheng S, Massaro JM, Fox CS (2010). Adiposity, cardiometabolic risk, and vitamin D status: the Framingham heart study. Diabetes.

[27] Wortsman J, Matsuoka LY, Chen TC, Lu Z, Holick MF (2000). Decreased bioavailability of vitamin D in obesity. Am J Clin Nutr.

[28] hmadieh H, Azar ST, Lakkis N, Arabi A. Hypovitaminosis d in patients with type 2 diabetes mellitus: a relation to disease control and complications. ISRN Endocrinology. 2013;2013:7 pages.641098. 10.1155/2013/641098 PMC3819758 24251044

[29] Drincic AT, Armas LAG, van Diest EE, Heaney RP (2012). Volumetric dilution, rather than sequestration best explains the low vitamin D status of obesity. Obesity.

[30] Major GC, Alarie F, Doré J, Phouttama S, Tremblay A (2007). Supplementation with calcium + vitamin D enhances the beneficial effect of weight loss on plasma lipid and lipoprotein concentrations. Am J Clin Nutr.

[31] Salehpour A, Hosseinpanah F, Shidfar F, Vafa M, Razaghi M, Dehghani S, Hoshiarrad A, Gohari M. A 12-week double-blind randomized clinical trial of vitamin D_3_ supplementation on body fat mass in healthy overweight and obese women. Nutr J. 2012;(22):11:78. 10.1186/1475-2891-11-78 PMC3514135 22998754

[32] Rock CL, Emond JA, Flatt SW, Heath DD, Karanja N, Pakiz B, Sherwood NE, Thomson CA (2012). Weight loss is associated with increased serum 25-hydroxyvitamin D in overweight or obese women. Obesity (Silver Spring).

[33] Swagerty DL, Walling AD, Klein RM (2002). Lactose intolerance. Am Fam Physician.

[34] Clemens TL, Adams JS, Henderson SL, Holick MF (1982). Increased skin pigment reduces the capacity of skin to synthesise vitamin D3. Lancet.

[35] Al-Timimi DJ, Ali AF (2013). Serum 25(OH) D in diabetes mellitus type 2: relation to Glycaemic control. J Clin Diagn Res.

[36] Chiu KC, Chu A, Go VL, Saad MF (2004). Hypovitaminosis D is associated with insulin resistance and beta cell dysfunction. Am J Clin Nutr.

[37] Ahmadieh H, Azar ST, Lakkis N, Arabi A (2013). Hypovitaminosis D in patients with type 2 diabetes mellitus: a relation to disease control and complications. ISRN Endocrinology.

[38] Huffman FG, De La Cera M, Vaccaro JA, Zarini GG, Exebio J, Gundupalli D, Shaban L (2011). Healthy eating index and alternate healthy eating index among Haitian Americans and African Americans with and without type 2 diabetes. J Nutr Metab.

[39] Désilets MC, Rivard M, Shatenstein B, Delisle H (2007). Dietary transition stages based on eating patterns and diet quality among Haitians of Montreal. Canada Public Health Nutr.

[40] Scott JM, McDougle L, Schwirian K, Taylor CA (2010). Differences in the dietary intake habits by diabetes status for African American adults. Ethnicity & Disease.

[41] Vimalananda VG, Rosenzweig JL, Cabral HJ, David MM, Lasser KE (2011). Comparison of diabetes control among Haitians, African Americans, and non-Hispanic whites in an urban safety-net hospital. Diabetes Care.

[42] Faridi KF, Zhao D, Martin SS, Lupton JR, Jones SR, Guallar E, Ballantyne CM, Lutsey PL, Michos ED. Serum vitamin D and change in lipid levels over 5 y: The Atherosclerosis Risk in Communities study. Nutrition. 2017;38:85-93. 10.1016/j.nut.2017.01.008 PMC5443111 28526388

[43] Wang Y, Si S, Liu J, Wang Z, Jia H, Feng K, Sun L, Song SJ (2016). The associations of serum lipids with Vitamin D status. PLoS One.

[44] Ford ES, Ajani UA, McGuire LC, Liu S (2005). Concentrations of serum vitamin D and the metabolic syndrome among U.S. adults. Diabetes Care.

[45] Karhapää P, Pihlajamäki J, Pörsti I, Kastarinen M, Mustonen J, Niemelä O, Kuusisto J (2010). Diverse associations of 25-hydroxyvitamin D and 1,25-dihydroxy-vitamin D with dyslipidaemias. J Intern Med.

[46] Auwerx J, Bouillon R, Kesteloot H (1992). Relation between 25-hydroxyvitamin D3, apolipoprotein A-I, and high density lipoprotein cholesterol. Arterioscler Thromb.

[47] Wang JH, Keisala T, Solakivi T, Minasyan A, Kalueff AV, Tuohimaa P (2009). Serum cholesterol and expression of ApoAI, LXR [beta] and SREBP2 in vitamin D receptor knock-out mice. The J Steroid Biochem.

[48] Christensen R, Lorenzen JK, Svith CR, Bartels E, Melanson E, Saris W, Tremblay A, Astrup A (2009). Effect of calcium from dairy and dietary supplements on faecal fat excretion: a meta-analysis of randomized controlled trials. Obes Rev.

[49] Husemoen LL, Skaaby T, Martinussen T, Jørgensen T, Thuesen BH, Kistorp C, Jeppesen J, Thyssen JP, Meldgaard M, Szecsi PB, Fenger M, Linneberg A (2014). Investigating the causal effect of vitamin D on serum adiponectin using a Mendelian randomization approach. Eur J Clin Nutr.

[50] Vaidya A, Williams JS, Forman JP (2012). The independent association between 25-hydroxyvitamin D and adiponectin and its relation with BMI in two large cohorts: the NHS and the HPFS. Obesity (Silver Spring).

[51] Vilarrasa N, Vendrell J, Maravall J, Elío I, Solano E, San José P, García I, Virgili N, Soler J, Gómez JM (2010). Is plasma 25(OH) D related to adipokines, inflammatory cytokines and insulin resistance in both a healthy and morbidly obese population?. Endocrine.

[52] Shea MK, Booth SL, Massaro JM, Jacques PF, D'Agostino RB, Dawson-Hughes B, Ordovas JM, O'Donnell CJ, Kathiresan S, Keaney JF, Vasan RS, Benjamin EJ, Vitamin K (2008). Vitamin D status: associations with inflammatory markers in the Framingham offspring study. Am J Epidemiol.

[53] Liu E, Meigs JB, Pittas AG, McKeown NM, Economos CD, Booth SL, Jacques PF (2009). Plasma 25-hydroxyvitamin d is associated with markers of the insulin resistant phenotype in nondiabetic adults. J Nutr.

[54] Bonora E, Targher G, Alberiche M, Bonadonna RC, Saggiani F, Zenere MB, Monauni T, Muggeo M (2000). Homeostasis model assessment closely mirrors the glucose clamp technique in the assessment of insulin sensitivity: studies in subjects with various degrees of glucose tolerance and insulin sensitivity. Diabetes Care.

