# Supplementary material for: Relationship of serum Vitamin D concentrations with Adipokines and Cardiometabolic risk among non-Hispanic black type 2 diabetic and non-diabetic subjects: a cross-sectional study
Source: BMC Nutr. 2018 Dec 18;4:50. doi: 10.1186/s40795-018-0259-2 (PMC7050721; doi:10.1186/s40795-018-0259-2)
Supplement: Supplementary file 1 — Haitian Americans and African Americans with T2D – (DOCX 12.3 kb) [file 40795_2018_259_MOESM1_ESM.docx]

**Additional File**

**Haitian Americans and African Americans with T2D –**

In MLR analyses within each ethnic subgroup for HA and AA with T2D, there were no significant associations between serum 25(OH)D concentrations and WC (R^2^ = 0.704, B = 0.009, *P* = 0.777), HbA1C (R^2^ = 0.057, B = 0.002, *P* = 0.907), and TG (R^2^ = 0.114, B = -0.165, *P* = 0.522) in only HA withT2D. While in only AA with T2D, serum 25(OH)D concentrations had no significant associations with TC (R^2^ = 0.037, B = -0.313, *P* = 0.118), log leptin (R^2^ = 0.393, B = -0.006, *P* = 0.075), and adiponectin (R^2^ = 0.170, B = -0.082, *P* = 0.329), after adjustment for previously stated covariates.
